# Crystal structure of μ-4-oxidobenzoato-κ^2^ *O* ^1^:*O* ^4^-bis­[bis­(1,10-phenanthroline-κ^2^ *N*,*N*′)copper(II)] bis­(4-hy­droxy­benzoate) 7.5-hydrate

**DOI:** 10.1107/S2056989016013943

**Published:** 2016-09-05

**Authors:** Jian-Rong Su, Yu Li

**Affiliations:** a School of Pharmaceutical and Chemical Engineering, Taizhou University, People’s Republic of China

**Keywords:** crystal structure, 4-hy­droxy­benzoate, 4-oxidobenzoate, 1,10-phenanthroline, copper, dinuclear complex cation

## Abstract

The title hydrated complex, [Cu_2_(C_7_H_4_O_3_)(C_12_H_8_N_2_)_4_](C_7_H_5_O_3_)_2_·7.5H_2_O, is composed of dinuclear Cu^II^ complex cations, noncoordinating 4-hy­droxy­benzoate anions and water mol­ecules of crystallization. In the dinuclear complex cation, the Cu^II^ ions are bridged by a 4-oxidobenzoate ligand and thus each metal ion is five-coordinated by two chelating 1,10-phenanthroline (phen) mol­ecules and one anionic O atom in a distorted trigonal-bipyramid geometry. In the crystal, aromatic π–π stacking occurs between phen rings of neighbouring dinuclear Cu^II^ complex cations, forming two-dimensional supra­molecular systems parallel to (100).

## Abstract 2

In the hydrated complex composed of dinuclear Cu^II^ complex cations, uncoordinated 4-hy­droxy­benzoate anions and water mol­ecules of crystallization, the Cu^II^ ions are bridged by a 4-oxidobenzoate ligand, each metal ion is five-coordinated by two chelated 1,10-phenanthroline (phen) mol­ecules and one anion O atom in distorted trigonal bipyramid geometry.

## Chemical context   

In some biological systems, π–π stacking between aromatic rings is correlated with the elctron-transfer process (Deisenhofer & Michel, 1989 ▸). To study the effect of π–π stacking, the title complex, (I), incorporating 1,10-phenanthroline (phen), has been prepared and its crystal structure is presented here.
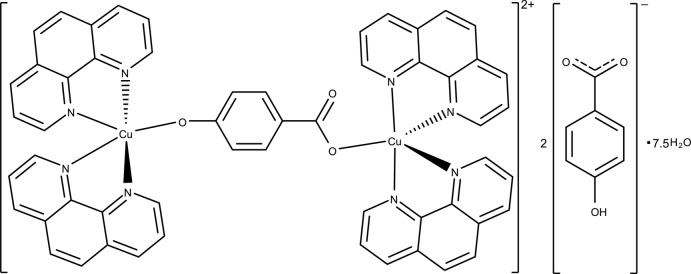


## Structural commentary   

**Figure 1:**
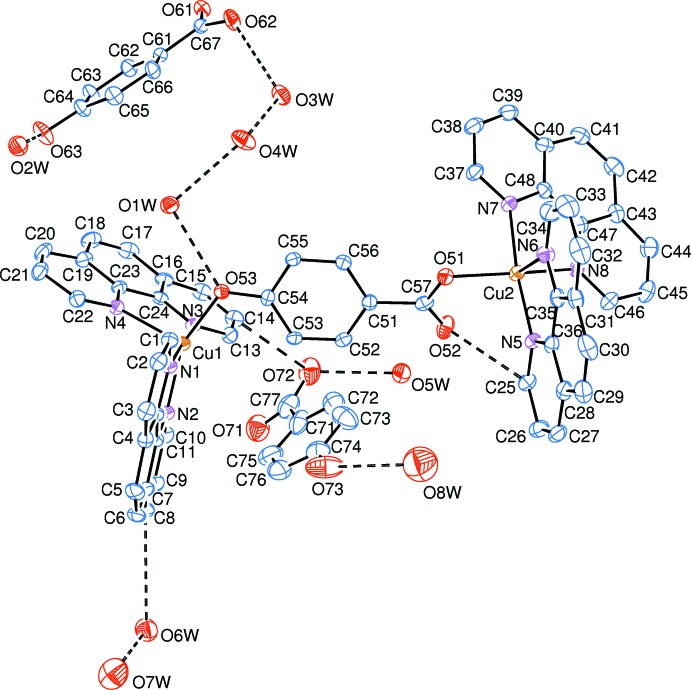
The structures of the molecular entities of (I), shown with 30% probability displacement ellipsoids. Dashed lines indicate hydrogen bonds. H atoms have been omitted for clarity.

**Figure 2:**
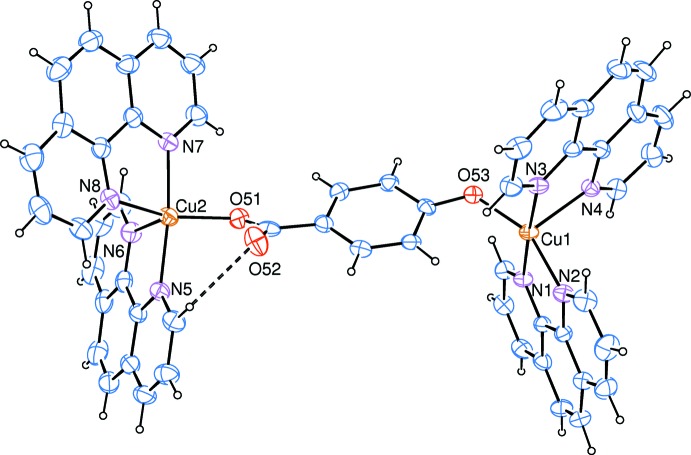
The mol­ecular structure of the dinuclear Cu^II^ complex cation in (I), shown with 30% probability displacement ellipsoids. H atoms have been omitted for clarity. Dashed lines indicate hydrogen bonds.

The crystal structure of (I) is composed of dinuclear Cu^II^ complex cations, noncoordinating 4-hy­droxy­benzoate anions and solvent water mol­ecules, as shown in Fig. 1 ▸. The mol­ecular structure of the dinuclear Cu^II^ complex cation is shown in Fig. 2 ▸. Two Cu^II^ atoms (Cu1 and Cu2) are bridged by one 4-oxidobenzoate anion through oxido and carb­oxy O atoms (O53 and O51, respectively), with a Cu1—O53 bond length of 1.941 (3) Å and a Cu2—O51 bond length of 1.979 (3) Å. Each Cu^II^ atom is five-coordinated, displaying a distorted trigonal-bipyramidal geometry (Table 1 ▸). The Cu1 atom is coordinated by two chelating phen rings (N1, N2, N3 and N4) inter­secting at an angle of 71.35 (5)°. The out-of-plane Cu1—N1 and Cu—N3 bond lengths are 2.002 (3) and 2.027 (3) Å, respectively, which are shorter than the in-plane Cu1—N2 and Cu1—N4 bond lengths [2.051 (4) and 2.158 (4) Å, respectively]. The N1—Cu1—N3 bond angle is 176.67 (15)°. The coordination parameters of the Cu2 atom are similar to those of the Cu1 atom. The Cu^II^ atoms display apparently different coordination patterns from the Cu^II^ complex coordinated by phen and 4-hy­droxy­benzoate ligands (Su *et al.*, 2005 ▸), in which the reported complex is mononuclear, with the Cu^II^ ion being six-coordinated by one chelating phen ligand and two chelating 4-hy­droxy­benzoate anions through the carb­oxylate O atoms, resulting in an elongated octa­hedral geometry.

## Supra­molecular features   

**Figure 3:**
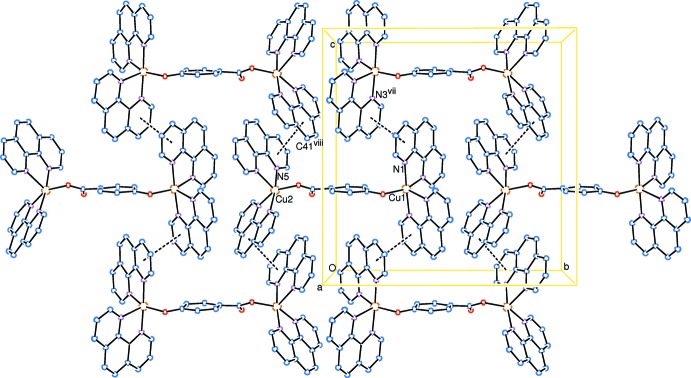
The π–π stacking (dashed lines) between neighbouring dinuclear Cu^II^ complex cations, forming a two-dimensional supra­molecular system parallel to (100). H atoms have been omitted for clarity. [Symmetry codes: (vii) *x*, −*y* + 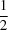, *z* + 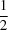; (viii) *x*, −*y* − 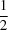, *z* + 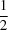.]

In the crystal of (I), π–π stacking inter­actions occur between neighbouring phen ligands and generate a two-dimensional supra­molecular system in the (100) plane, as shown in Fig. 3 ▸. The N1- and N3^vii^-phen [symmetry code: (vii) *x*, −*y* + 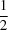, *z* + 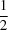] ligands are nearly parallel, the dihedral angle being 5.00 (11)° and the shortest distance between the centroids of the aromatic rings (N1- and N3^vii^-rings) being 3.647 (3) Å. These findings indicate π–π stacking between the N1- and N3^vii^-phen ligands of neighbouring complexes. The same is true for the N5- and C41^viii^-phen [symmetry code: (viii) *x*, −*y* − 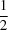, *z* + 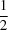] ligands, the dihedral angle being 8.48 (13)° and the shortest distance between the centroids of the aromatic rings (N5- and C41^viii^-rings) being 3.671 (3) Å.

**Figure 4:**
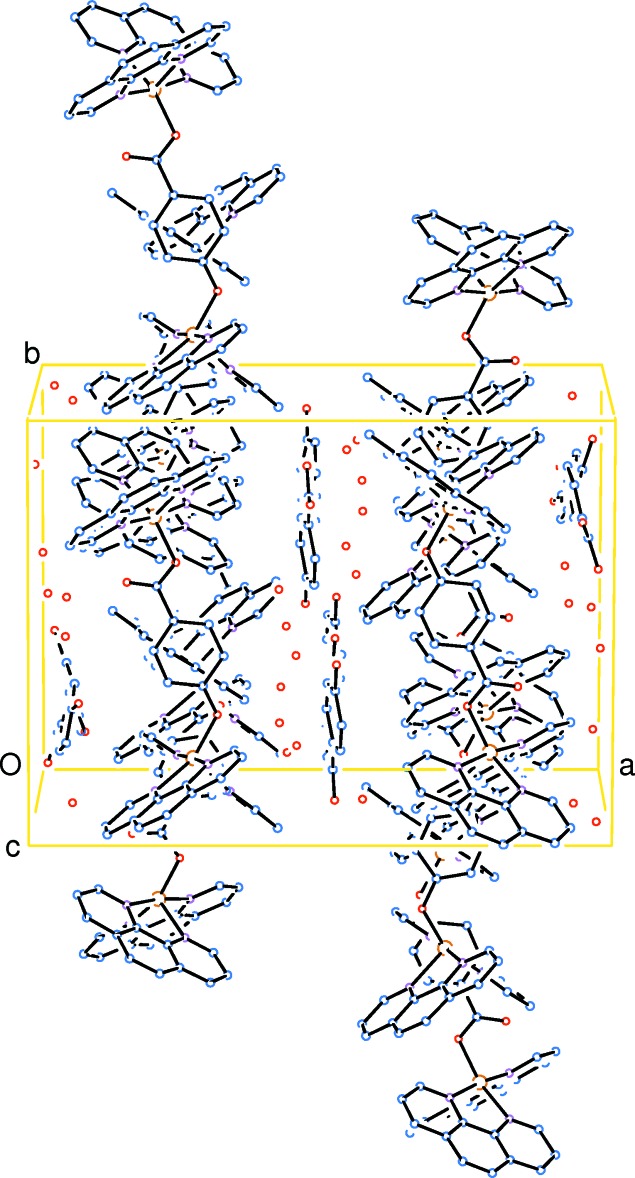
A packing diagram showing alternating layers along the *a* axis, one layer consisting of dinuclear Cu^II^ complex cations, the other consisting of noncoordinating 4-hydroxybenzoate dianions and solvent water mol­ecules. H atoms have been omitted for clarity.

The mol­ecular packing of (I), as shown in Fig. 4 ▸, displays alternating layers along the *a* axis, one layer consisting of dinuclear Cu^II^ complex cations (complex-layer), the other consisting of noncoordinating 4-hy­droxy­benzoate anions and solvent water mol­ecules (solvent-layer). Abundant hydrogen-bonding inter­actions occur within the solvent-layer and among the solvent- and complex-layers (Table 2 ▸). The H atoms on O6*W*, O7*W* and O8*W* were not assigned in the structure, the separations [O6*W*⋯O7*W* = 2.729 (8) Å and O8*W*⋯O73 2.88 (2) Å] suggest inter­molecular hydrogen bonding between atoms O6*W* and O7*W*, and between O8*W* and O73.

## Synthesis and crystallization   

Each reagent was available commercially and was of analytical grade. CuCl_2_·2H_2_O (0.17 g, 1 mmol), 4-hy­droxy­benzoic acid (0.28 g, 2 mmol), 1,10-phenanthroline (0.20 g, 1 mmol) and NaOH (0.16 g, 4 mmol) were dissolved in 20 ml water. The resulting solution was refluxed for 4 h and was then cooled to room temperature and filtered. Dark-green single crystals were obtained from the filtrate after five weeks.

## Refinement   

Crystal data, data collection and structure refinement details are summarized in Table 3 ▸. The disordered water O8*W* atom was refined isotropically with a fixed occupacy of 0.5. Aromatic and hy­droxy H atoms were placed in calculated positions, with C—H = 0.93 Å and O—H = 0.82 Å, and were included in the final cycles of refinement in riding mode, with *U*_iso_(H) = 1.2 and 1.5*U*_eq_(parent), respectively. Water H atoms were located in difference Fourier map, and were refined with fixed positions and a fixed isotropic displacement parameter of 0.1 Å^2^. The H atoms of the water molecules O6*W*, O7*W* and O8*W* were not assigned. The peak corresponding to the maximum electron density in the difference Fourier map was close (1.01 Å) to atom O8*W*.

## Supplementary Material

Crystal structure: contains datablock(s) I, global. DOI: 10.1107/S2056989016013943/hb7601sup1.cif

Structure factors: contains datablock(s) I. DOI: 10.1107/S2056989016013943/hb7601Isup2.hkl

CCDC reference: 1501982

Additional supporting information: crystallographic information; 3D view; checkCIF report

## Figures and Tables

**Table 1:** Selected geometric parameters (Å, °)

Cu1—O53	1.941 (3)	Cu2—O51	1.979 (3)
Cu1—N1	2.002 (3)	Cu2—N5	2.010 (4)
Cu1—N2	2.051 (4)	Cu2—N6	2.197 (4)
Cu1—N3	2.027 (3)	Cu2—N7	2.013 (4)
Cu1—N4	2.158 (4)	Cu2—N8	2.079 (4)
			
N1—Cu1—N3	176.67 (15)	N5—Cu2—N7	173.07 (16)
O53—Cu1—N2	155.19 (14)	O51—Cu2—N5	96.10 (15)
O53—Cu1—N4	101.39 (14)	O51—Cu2—N7	89.77 (15)
N2—Cu1—N4	103.05 (15)	O51—Cu2—N8	152.80 (15)
O53—Cu1—N1	89.92 (14)	N5—Cu2—N8	94.96 (16)
O53—Cu1—N3	93.37 (14)	N7—Cu2—N8	81.32 (17)
N1—Cu1—N2	81.74 (14)	O51—Cu2—N6	101.29 (16)
N3—Cu1—N2	95.05 (15)	N5—Cu2—N6	79.62 (16)
N1—Cu1—N4	100.08 (14)	N7—Cu2—N6	95.64 (16)
N3—Cu1—N4	79.74 (15)	N8—Cu2—N6	105.12 (16)

**Table 2:** Hydrogen-bond geometry (Å, °)

*D*—H⋯*A*	*D*—H	H⋯*A*	*D*⋯*A*	*D*—H⋯*A*
O1*W*—H1*A*⋯O53	0.97	1.90	2.819 (5)	157
O1*W*—H1*B*⋯O61^i^	1.00	1.76	2.758 (5)	169
O2*W*—H2*A*⋯O61^i^	0.97	1.75	2.708 (5)	169
O2*W*—H2*B*⋯O4*W* ^ii^	0.83	2.04	2.843 (6)	160
O3*W*—H3*A*⋯O62^iii^	1.00	1.69	2.685 (5)	172
O3*W*—H3*B*⋯O62	1.00	1.84	2.731 (7)	147
O4*W*—H4*A*⋯O1*W*	1.01	1.79	2.756 (6)	160
O4*W*—H4*B*⋯O3*W*	0.86	1.94	2.750 (7)	157
O5*W*—H5*A*⋯O72	0.96	1.87	2.818 (8)	169
O5*W*—H5*B*⋯O6*W* ^iv^	0.97	1.84	2.779 (7)	164
O73—H73⋯O8*W*	0.82	2.15	2.88 (2)	148
O63—H63⋯O2*W*	0.82	1.85	2.638 (5)	161
C3—H3⋯O1*W* ^i^	0.93	2.59	3.240 (6)	127
C8—H8⋯O6*W*	0.93	2.48	3.371 (8)	162
C14—H14⋯O72	0.93	2.50	3.388 (10)	159
C21—H21⋯O4*W* ^ii^	0.93	2.58	3.230 (8)	128
C25—H25⋯O52	0.93	2.47	3.033 (6)	119
C33—H33⋯O62^v^	0.93	2.55	3.247 (8)	132
C38—H38⋯O61^iii^	0.93	2.37	3.298 (8)	172
C65—H65⋯O63^vi^	0.93	2.49	3.410 (6)	172
C73—H71⋯O7*W* ^iv^	0.93	2.58	3.413 (13)	149

Symmetry codes: (i) 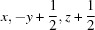; (ii) 
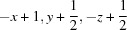
; (iii) 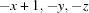; (iv) 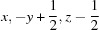; (v) 
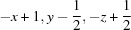
; (vi) 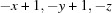.

**Table 3:** Experimental details

Crystal data	
Chemical formula	[Cu_2_(C_7_H_4_O_3_)(C_12_H_8_N_2_)_4_](C_7_H_5_O_3_)_2_·7.5H_2_O
*M*_r_	1393.33
Crystal system, space group	Monoclinic, *P*2_1_/*c*
Temperature (K)	296
*a*, *b*, *c* (Å)	22.6830 (11), 16.6644 (6), 16.8388 (6)
β (°)	91.026 (3)
*V* (Å^3^)	6364.0 (4)
*Z*	4
Radiation type	Mo *K*α
μ (mm^−1^)	0.75
Crystal size (mm)	0.45 × 0.40 × 0.30

Data collection	
Diffractometer	Xcalibur Atlas Gemini ultra
Absorption correction	Multi-scan (*CrysAlis PRO*; Agilent, 2014 ▸)
*T*_min_, *T*_max_	0.929, 1.000
No. of measured, independent and observed [*I* > 2σ(*I*)] reflections	27049, 11598, 7651
*R*_int_	0.048
(sin θ/λ)_max_ (Å^−1^)	0.602

Refinement	
*R*[*F* ^2^ > 2σ(*F* ^2^)], *wR*(*F* ^2^), *S*	0.067, 0.201, 1.03
No. of reflections	11598
No. of parameters	861
H-atom treatment	H atoms treated by a mixture of independent and constrained refinement
Δρ_max_, Δρ_min_ (e Å^−3^)	0.90, −0.56

Computer programs: *CrysAlis PRO* (Agilent, 2014 ▸), *SIR92* (Altomare *et al.*, 1994 ▸), *SHELXL2014* (Sheldrick, 2015 ▸) and *ORTEP-3 for Windows* and *WinGX* (Farrugia, 2012 ▸).

## supplementary crystallographic information

### Crystal data

**Table:**

[Cu_2_(C_7_H_4_O_3_)(C_12_H_8_N_2_)_4_](C_7_H_5_O_3_)_2_·7.5H_2_O	*F*(000) = 2884
*M**_r_* = 1393.33	*D*_x_ = 1.454 Mg m^−^^3^
Monoclinic, *P*2_1_/*c*	Mo *K*α radiation, λ = 0.71073 Å
*a* = 22.6830 (11) Å	Cell parameters from 5425 reflections
*b* = 16.6644 (6) Å	θ = 3.2–29.4°
*c* = 16.8388 (6) Å	µ = 0.75 mm^−^^1^
β = 91.026 (3)°	*T* = 296 K
*V* = 6364.0 (4) Å^3^	Block, dark green
*Z* = 4	0.45 × 0.40 × 0.30 mm

### Data collection

**Table:**

Xcalibur Atlas Gemini ultra diffractometer	11598 independent reflections
Radiation source: Enhance (Mo) X-ray Source	7651 reflections with *I* > 2σ(*I*)
Detector resolution: 10.3592 pixels mm^-1^	*R*_int_ = 0.048
ω scans	θ_max_ = 25.4°, θ_min_ = 3.2°
Absorption correction: multi-scan (CrysAlis PRO; Agilent, 2014)	*h* = −27→24
*T*_min_ = 0.929, *T*_max_ = 1.000	*k* = −16→20
27049 measured reflections	*l* = −16→20

### Refinement

**Table:**

Refinement on *F*^2^	0 restraints
Least-squares matrix: full	Hydrogen site location: mixed
*R*[*F*^2^ > 2σ(*F*^2^)] = 0.067	H atoms treated by a mixture of independent and constrained refinement
*wR*(*F*^2^) = 0.201	*w* = 1/[σ^2^(*F*_o_^2^) + (0.0916*P*)^2^ + 7.0203*P*] where *P* = (*F*_o_^2^ + 2*F*_c_^2^)/3
*S* = 1.03	(Δ/σ)_max_ < 0.001
11598 reflections	Δρ_max_ = 0.90 e Å^−^^3^
861 parameters	Δρ_min_ = −0.56 e Å^−^^3^

### Special details

**Table:**

Experimental. Absorption correction: CrysAlis PRO, Agilent Technologies, Version 1.171.35.11 (release 16-05-2011 CrysAlis171 .NET) (compiled May 16 2011,17:55:39) Empirical absorption correction using spherical harmonics, implemented in SCALE3 ABSPACK scaling algorithm.
Geometry. All esds (except the esd in the dihedral angle between two l.s. planes) are estimated using the full covariance matrix. The cell esds are taken into account individually in the estimation of esds in distances, angles and torsion angles; correlations between esds in cell parameters are only used when they are defined by crystal symmetry. An approximate (isotropic) treatment of cell esds is used for estimating esds involving l.s. planes.

### Fractional atomic coordinates and isotropic or equivalent isotropic displacement parameters (Å^2^)

**Table:**

	*x*	*y*	*z*	*U*_iso_*/*U*_eq_	Occ. (<1)
Cu1	0.28079 (2)	0.31771 (3)	0.36174 (3)	0.03912 (18)	
Cu2	0.21227 (3)	−0.25364 (4)	0.35545 (3)	0.04569 (19)	
O51	0.25057 (16)	−0.1490 (2)	0.3772 (2)	0.0555 (9)	
O52	0.16596 (18)	−0.0964 (2)	0.3379 (2)	0.0705 (11)	
O53	0.32159 (14)	0.21783 (19)	0.34046 (17)	0.0449 (8)	
C51	0.2467 (2)	−0.0085 (3)	0.3632 (3)	0.0442 (11)	
C52	0.2127 (2)	0.0605 (3)	0.3706 (3)	0.0453 (11)	
H52	0.1723	0.0554	0.3776	0.054*	
C53	0.2368 (2)	0.1362 (3)	0.3678 (2)	0.0423 (11)	
H53	0.2130	0.1807	0.3762	0.051*	
C54	0.2969 (2)	0.1473 (3)	0.3527 (2)	0.0403 (11)	
C55	0.3326 (2)	0.0770 (3)	0.3476 (3)	0.0450 (11)	
H55	0.3729	0.0818	0.3403	0.054*	
C56	0.3070 (2)	0.0013 (3)	0.3535 (3)	0.0474 (12)	
H56	0.3310	−0.0439	0.3509	0.057*	
C57	0.2179 (2)	−0.0904 (3)	0.3594 (2)	0.0468 (12)	
N1	0.30647 (16)	0.3111 (2)	0.4759 (2)	0.0388 (9)	
N2	0.21591 (17)	0.3878 (2)	0.4091 (2)	0.0425 (9)	
N3	0.25177 (17)	0.3299 (2)	0.2478 (2)	0.0416 (9)	
N4	0.34316 (17)	0.4048 (2)	0.3191 (2)	0.0409 (9)	
N5	0.15784 (18)	−0.2504 (2)	0.4483 (2)	0.0439 (9)	
N6	0.2601 (2)	−0.3322 (2)	0.4393 (2)	0.0531 (11)	
N7	0.26698 (18)	−0.2714 (2)	0.2643 (2)	0.0471 (10)	
N8	0.15934 (19)	−0.3302 (2)	0.2877 (2)	0.0475 (10)	
C1	0.3531 (2)	0.2736 (3)	0.5068 (3)	0.0452 (11)	
H1	0.3779	0.2456	0.4732	0.054*	
C2	0.3660 (2)	0.2747 (3)	0.5880 (3)	0.0480 (12)	
H2	0.3990	0.2477	0.6079	0.058*	
C3	0.3305 (2)	0.3153 (3)	0.6377 (3)	0.0517 (13)	
H3	0.3391	0.3161	0.6919	0.062*	
C4	0.2808 (2)	0.3563 (3)	0.6076 (3)	0.0454 (11)	
C5	0.2391 (3)	0.3988 (3)	0.6544 (3)	0.0596 (15)	
H5	0.2452	0.4020	0.7091	0.071*	
C6	0.1913 (3)	0.4342 (3)	0.6214 (3)	0.0579 (14)	
H6	0.1642	0.4593	0.6539	0.069*	
C7	0.1815 (2)	0.4341 (3)	0.5370 (3)	0.0477 (12)	
C8	0.1348 (2)	0.4741 (3)	0.4984 (4)	0.0622 (15)	
H8	0.1073	0.5028	0.5273	0.075*	
C9	0.1304 (3)	0.4703 (4)	0.4179 (4)	0.0681 (16)	
H9	0.0999	0.4970	0.3913	0.082*	
C10	0.1714 (2)	0.4265 (3)	0.3746 (3)	0.0560 (13)	
H10	0.1673	0.4244	0.3196	0.067*	
C11	0.2211 (2)	0.3924 (3)	0.4898 (3)	0.0389 (10)	
C12	0.2711 (2)	0.3523 (3)	0.5256 (2)	0.0380 (10)	
C13	0.2056 (2)	0.2941 (3)	0.2141 (3)	0.0513 (12)	
H13	0.1817	0.2621	0.2453	0.062*	
C14	0.1915 (3)	0.3025 (4)	0.1335 (3)	0.0605 (14)	
H14	0.1587	0.2767	0.1116	0.073*	
C15	0.2262 (3)	0.3486 (4)	0.0876 (3)	0.0611 (15)	
H15	0.2168	0.3551	0.0340	0.073*	
C16	0.2755 (2)	0.3862 (3)	0.1196 (3)	0.0510 (13)	
C17	0.3165 (3)	0.4348 (4)	0.0758 (3)	0.0648 (16)	
H17	0.3104	0.4412	0.0214	0.078*	
C18	0.3629 (3)	0.4709 (4)	0.1108 (3)	0.0683 (17)	
H18	0.3889	0.5004	0.0801	0.082*	
C19	0.3732 (2)	0.4649 (3)	0.1948 (3)	0.0546 (13)	
C20	0.4194 (3)	0.5043 (3)	0.2362 (4)	0.0666 (16)	
H20	0.4452	0.5376	0.2093	0.080*	
C21	0.4259 (2)	0.4930 (3)	0.3147 (4)	0.0627 (15)	
H21	0.4561	0.5189	0.3426	0.075*	
C22	0.3875 (2)	0.4429 (3)	0.3546 (3)	0.0509 (12)	
H22	0.3933	0.4357	0.4089	0.061*	
C23	0.3358 (2)	0.4162 (3)	0.2396 (3)	0.0416 (11)	
C24	0.2863 (2)	0.3766 (3)	0.2021 (2)	0.0422 (11)	
C25	0.1075 (2)	−0.2109 (3)	0.4523 (3)	0.0552 (13)	
H25	0.0948	−0.1815	0.4083	0.066*	
C26	0.0724 (3)	−0.2114 (4)	0.5202 (3)	0.0637 (15)	
H26	0.0372	−0.1828	0.5208	0.076*	
C27	0.0901 (3)	−0.2538 (4)	0.5846 (3)	0.0639 (16)	
H27	0.0671	−0.2548	0.6297	0.077*	
C28	0.1432 (3)	−0.2959 (3)	0.5833 (3)	0.0558 (14)	
C29	0.1664 (3)	−0.3405 (4)	0.6497 (3)	0.0697 (18)	
H29	0.1449	−0.3437	0.6960	0.084*	
C30	0.2190 (4)	−0.3780 (4)	0.6459 (3)	0.0734 (19)	
H30	0.2337	−0.4047	0.6906	0.088*	
C31	0.2526 (3)	−0.3777 (3)	0.5754 (3)	0.0567 (14)	
C32	0.3070 (3)	−0.4161 (4)	0.5678 (4)	0.079 (2)	
H32	0.3233	−0.4443	0.6106	0.095*	
C33	0.3362 (3)	−0.4122 (4)	0.4980 (5)	0.082 (2)	
H33	0.3725	−0.4374	0.4925	0.098*	
C34	0.3107 (3)	−0.3700 (4)	0.4355 (4)	0.0733 (17)	
H34	0.3307	−0.3684	0.3878	0.088*	
C35	0.2311 (2)	−0.3354 (3)	0.5093 (3)	0.0482 (12)	
C36	0.1758 (2)	−0.2933 (3)	0.5137 (3)	0.0470 (12)	
C37	0.3202 (2)	−0.2411 (3)	0.2541 (3)	0.0568 (14)	
H37	0.3362	−0.2074	0.2929	0.068*	
C38	0.3530 (3)	−0.2579 (4)	0.1875 (4)	0.0665 (16)	
H38	0.3904	−0.2359	0.1826	0.080*	
C39	0.3308 (3)	−0.3062 (4)	0.1292 (4)	0.0655 (15)	
H39	0.3530	−0.3175	0.0847	0.079*	
C40	0.2741 (3)	−0.3392 (3)	0.1368 (3)	0.0550 (13)	
C41	0.2453 (3)	−0.3883 (3)	0.0788 (3)	0.0635 (15)	
H41	0.2637	−0.3984	0.0309	0.076*	
C42	0.1923 (3)	−0.4201 (4)	0.0919 (3)	0.0692 (17)	
H42	0.1755	−0.4536	0.0536	0.083*	
C43	0.1599 (3)	−0.4042 (3)	0.1637 (3)	0.0569 (14)	
C44	0.1043 (3)	−0.4333 (4)	0.1801 (4)	0.0706 (17)	
H44	0.0855	−0.4680	0.1447	0.085*	
C45	0.0771 (3)	−0.4111 (4)	0.2480 (4)	0.0736 (18)	
H45	0.0397	−0.4304	0.2594	0.088*	
C46	0.1062 (2)	−0.3589 (3)	0.3002 (3)	0.0599 (14)	
H46	0.0872	−0.3436	0.3462	0.072*	
C47	0.1866 (2)	−0.3534 (3)	0.2202 (3)	0.0461 (11)	
C48	0.2441 (2)	−0.3208 (3)	0.2068 (3)	0.0470 (12)	
O61	0.51842 (17)	0.1737 (2)	−0.1530 (2)	0.0586 (9)	
O62	0.5244 (2)	0.0993 (2)	−0.0439 (2)	0.0754 (12)	
O63	0.5268 (2)	0.4457 (2)	0.1098 (2)	0.0846 (14)	
H63	0.5352	0.4351	0.1561	0.127*	
C61	0.5215 (2)	0.2393 (3)	−0.0278 (3)	0.0455 (11)	
C62	0.5337 (3)	0.2353 (3)	0.0539 (3)	0.0571 (14)	
H62	0.5405	0.1854	0.0771	0.068*	
C63	0.5359 (3)	0.3023 (3)	0.1005 (3)	0.0603 (14)	
H63A	0.5447	0.2980	0.1545	0.072*	
C64	0.5248 (3)	0.3771 (3)	0.0665 (3)	0.0582 (14)	
C65	0.5116 (3)	0.3817 (3)	−0.0132 (3)	0.0624 (15)	
H65	0.5036	0.4314	−0.0361	0.075*	
C66	0.5101 (3)	0.3140 (3)	−0.0599 (3)	0.0553 (13)	
H66	0.5012	0.3186	−0.1138	0.066*	
C67	0.5211 (2)	0.1660 (3)	−0.0794 (3)	0.0497 (12)	
O73	0.0847 (3)	0.1727 (5)	0.4492 (4)	0.159 (3)	
H73	0.0901	0.1240	0.4488	0.238*	
O71	0.0240 (3)	0.3687 (4)	0.1367 (4)	0.135 (2)	
O72	0.0528 (3)	0.2498 (4)	0.0767 (5)	0.125 (2)	
C71	0.0508 (3)	0.2611 (5)	0.2203 (7)	0.104 (3)	
C72	0.0645 (4)	0.1836 (6)	0.2302 (6)	0.110 (3)	
H72	0.0674	0.1508	0.1858	0.132*	
C73	0.0747 (4)	0.1501 (6)	0.3063 (6)	0.121 (3)	
H71	0.0830	0.0956	0.3114	0.145*	
C74	0.0723 (4)	0.1985 (6)	0.3739 (7)	0.110 (3)	
C75	0.0613 (4)	0.2808 (7)	0.3649 (7)	0.118 (3)	
H75	0.0622	0.3146	0.4089	0.141*	
C76	0.0485 (4)	0.3131 (7)	0.2862 (6)	0.115 (3)	
H76	0.0390	0.3670	0.2795	0.138*	
C77	0.0414 (4)	0.2948 (7)	0.1392 (6)	0.109 (3)	
O1W	0.42849 (17)	0.2505 (2)	0.2640 (2)	0.0635 (10)	
O2W	0.5730 (2)	0.4352 (2)	0.2543 (2)	0.0740 (11)	
O3W	0.4390 (2)	0.0531 (2)	0.0588 (3)	0.0781 (12)	
O4W	0.4567 (2)	0.0978 (2)	0.2146 (2)	0.0856 (14)	
O5W	−0.0116 (2)	0.1084 (3)	0.0453 (3)	0.0922 (14)	
O6W	0.0242 (2)	0.5351 (3)	0.6151 (3)	0.1036 (16)	
O7W	0.0604 (3)	0.5505 (5)	0.7698 (4)	0.153 (3)	
O8W	0.0634 (10)	0.0074 (14)	0.4893 (13)	0.227 (8)*	0.5
H1A	0.3987	0.2333	0.3013	0.100*	
H1B	0.4602	0.2736	0.2996	0.100*	
H2A	0.5517	0.4018	0.2909	0.100*	
H2B	0.5714	0.4851	0.2567	0.100*	
H3A	0.4549	−0.0030	0.0576	0.100*	
H3B	0.4725	0.0848	0.0377	0.100*	
H4A	0.4564	0.1556	0.2317	0.100*	
H4B	0.4475	0.0970	0.1650	0.100*	
H5A	0.0137	0.1524	0.0598	0.100*	
H5B	0.0042	0.0564	0.0602	0.100*	

### Atomic displacement parameters (Å^2^)

**Table:**

	*U*^11^	*U*^22^	*U*^33^	*U*^12^	*U*^13^	*U*^23^
Cu1	0.0415 (3)	0.0474 (3)	0.0285 (3)	0.0009 (2)	−0.0005 (2)	−0.0004 (2)
Cu2	0.0418 (4)	0.0512 (4)	0.0442 (3)	−0.0054 (3)	0.0042 (3)	0.0018 (3)
O51	0.054 (2)	0.053 (2)	0.059 (2)	−0.0086 (18)	0.0087 (17)	−0.0072 (18)
O52	0.061 (3)	0.074 (3)	0.076 (3)	−0.020 (2)	−0.023 (2)	0.018 (2)
O53	0.049 (2)	0.0449 (18)	0.0413 (17)	−0.0024 (15)	0.0074 (14)	−0.0023 (15)
C51	0.052 (3)	0.046 (3)	0.034 (2)	−0.006 (2)	0.004 (2)	0.000 (2)
C52	0.039 (3)	0.060 (3)	0.038 (2)	−0.002 (2)	0.005 (2)	0.002 (2)
C53	0.044 (3)	0.049 (3)	0.035 (2)	0.001 (2)	0.0104 (19)	−0.002 (2)
C54	0.052 (3)	0.048 (3)	0.0215 (19)	−0.014 (2)	−0.0012 (18)	−0.0006 (19)
C55	0.037 (3)	0.050 (3)	0.047 (3)	−0.003 (2)	0.009 (2)	−0.006 (2)
C56	0.050 (3)	0.047 (3)	0.045 (3)	−0.001 (2)	0.006 (2)	−0.006 (2)
C57	0.048 (3)	0.070 (3)	0.023 (2)	−0.001 (3)	0.004 (2)	0.001 (2)
N1	0.037 (2)	0.049 (2)	0.0297 (18)	0.0019 (17)	−0.0010 (15)	−0.0002 (16)
N2	0.040 (2)	0.053 (2)	0.0348 (19)	0.0025 (18)	−0.0009 (16)	0.0042 (18)
N3	0.041 (2)	0.054 (2)	0.0300 (18)	0.0006 (18)	0.0007 (16)	−0.0032 (17)
N4	0.043 (2)	0.045 (2)	0.0343 (19)	−0.0016 (18)	−0.0009 (16)	0.0006 (17)
N5	0.038 (2)	0.050 (2)	0.044 (2)	−0.0034 (18)	0.0007 (17)	0.0018 (18)
N6	0.053 (3)	0.053 (2)	0.053 (2)	0.005 (2)	−0.001 (2)	0.004 (2)
N7	0.045 (2)	0.052 (2)	0.045 (2)	−0.0036 (19)	0.0054 (18)	0.0035 (19)
N8	0.049 (3)	0.046 (2)	0.048 (2)	−0.0048 (19)	−0.0017 (19)	0.0075 (19)
C1	0.042 (3)	0.048 (3)	0.045 (3)	0.004 (2)	−0.002 (2)	0.001 (2)
C2	0.041 (3)	0.056 (3)	0.047 (3)	−0.002 (2)	−0.009 (2)	0.007 (2)
C3	0.059 (3)	0.064 (3)	0.032 (2)	−0.009 (3)	−0.007 (2)	0.008 (2)
C4	0.055 (3)	0.048 (3)	0.033 (2)	−0.008 (2)	0.000 (2)	0.004 (2)
C5	0.085 (4)	0.063 (3)	0.031 (2)	0.000 (3)	0.012 (3)	−0.005 (2)
C6	0.067 (4)	0.054 (3)	0.053 (3)	0.006 (3)	0.023 (3)	−0.009 (3)
C7	0.048 (3)	0.048 (3)	0.048 (3)	0.000 (2)	0.012 (2)	0.001 (2)
C8	0.050 (3)	0.058 (3)	0.079 (4)	0.011 (3)	0.011 (3)	−0.004 (3)
C9	0.051 (4)	0.069 (4)	0.085 (4)	0.019 (3)	−0.008 (3)	0.002 (3)
C10	0.052 (3)	0.066 (3)	0.050 (3)	0.004 (3)	−0.008 (2)	0.005 (3)
C11	0.039 (3)	0.039 (2)	0.039 (2)	−0.003 (2)	0.0034 (19)	−0.003 (2)
C12	0.040 (3)	0.040 (2)	0.034 (2)	−0.005 (2)	0.0043 (19)	−0.003 (2)
C13	0.051 (3)	0.061 (3)	0.041 (3)	−0.008 (3)	−0.005 (2)	−0.006 (2)
C14	0.057 (4)	0.077 (4)	0.047 (3)	0.007 (3)	−0.013 (3)	−0.011 (3)
C15	0.071 (4)	0.079 (4)	0.033 (3)	0.016 (3)	−0.015 (3)	−0.005 (3)
C16	0.058 (3)	0.060 (3)	0.035 (2)	0.016 (3)	0.005 (2)	0.001 (2)
C17	0.085 (5)	0.071 (4)	0.039 (3)	0.023 (3)	0.010 (3)	0.020 (3)
C18	0.074 (4)	0.068 (4)	0.064 (4)	0.007 (3)	0.026 (3)	0.030 (3)
C19	0.052 (3)	0.049 (3)	0.063 (3)	0.007 (2)	0.010 (3)	0.019 (3)
C20	0.047 (3)	0.060 (3)	0.093 (5)	−0.006 (3)	0.018 (3)	0.024 (3)
C21	0.045 (3)	0.063 (3)	0.080 (4)	−0.010 (3)	−0.001 (3)	0.005 (3)
C22	0.046 (3)	0.053 (3)	0.053 (3)	0.000 (2)	−0.005 (2)	0.000 (2)
C23	0.040 (3)	0.040 (2)	0.044 (3)	0.008 (2)	0.005 (2)	0.008 (2)
C24	0.050 (3)	0.045 (3)	0.032 (2)	0.012 (2)	0.002 (2)	0.001 (2)
C25	0.045 (3)	0.066 (3)	0.056 (3)	−0.005 (3)	−0.001 (2)	0.004 (3)
C26	0.047 (3)	0.082 (4)	0.062 (4)	−0.008 (3)	0.007 (3)	−0.008 (3)
C27	0.061 (4)	0.078 (4)	0.053 (3)	−0.025 (3)	0.018 (3)	−0.013 (3)
C28	0.073 (4)	0.056 (3)	0.038 (3)	−0.021 (3)	0.002 (2)	−0.007 (2)
C29	0.115 (6)	0.058 (3)	0.037 (3)	−0.019 (4)	0.007 (3)	−0.002 (3)
C30	0.125 (6)	0.053 (3)	0.042 (3)	−0.001 (4)	−0.018 (3)	−0.002 (3)
C31	0.081 (4)	0.042 (3)	0.046 (3)	−0.007 (3)	−0.018 (3)	0.001 (2)
C32	0.101 (6)	0.062 (4)	0.074 (4)	0.006 (4)	−0.038 (4)	0.003 (3)
C33	0.069 (4)	0.077 (4)	0.098 (5)	0.023 (4)	−0.026 (4)	0.002 (4)
C34	0.058 (4)	0.076 (4)	0.085 (4)	0.010 (3)	−0.005 (3)	0.010 (3)
C35	0.053 (3)	0.041 (3)	0.050 (3)	−0.007 (2)	−0.010 (2)	−0.004 (2)
C36	0.058 (3)	0.045 (3)	0.038 (2)	−0.013 (2)	0.001 (2)	−0.001 (2)
C37	0.043 (3)	0.065 (3)	0.062 (3)	−0.009 (3)	0.004 (3)	−0.001 (3)
C38	0.051 (4)	0.077 (4)	0.072 (4)	−0.003 (3)	0.016 (3)	0.008 (3)
C39	0.062 (4)	0.068 (4)	0.067 (4)	0.009 (3)	0.021 (3)	0.006 (3)
C40	0.058 (4)	0.057 (3)	0.050 (3)	0.015 (3)	0.008 (2)	0.002 (3)
C41	0.075 (4)	0.062 (3)	0.054 (3)	0.012 (3)	0.005 (3)	−0.003 (3)
C42	0.088 (5)	0.068 (4)	0.052 (3)	0.009 (3)	−0.013 (3)	−0.011 (3)
C43	0.059 (4)	0.049 (3)	0.062 (3)	0.002 (3)	−0.015 (3)	0.000 (3)
C44	0.067 (4)	0.072 (4)	0.072 (4)	−0.014 (3)	−0.017 (3)	−0.007 (3)
C45	0.056 (4)	0.077 (4)	0.086 (4)	−0.027 (3)	−0.011 (3)	0.008 (4)
C46	0.049 (3)	0.070 (4)	0.061 (3)	−0.010 (3)	0.005 (3)	0.009 (3)
C47	0.052 (3)	0.042 (3)	0.044 (3)	0.002 (2)	−0.005 (2)	0.006 (2)
C48	0.054 (3)	0.041 (3)	0.046 (3)	0.005 (2)	−0.002 (2)	0.007 (2)
O61	0.064 (3)	0.052 (2)	0.059 (2)	0.0041 (18)	−0.0006 (18)	−0.0072 (18)
O62	0.106 (4)	0.038 (2)	0.083 (3)	0.005 (2)	0.003 (2)	0.002 (2)
O63	0.138 (4)	0.050 (2)	0.066 (3)	0.016 (2)	−0.007 (2)	−0.010 (2)
C61	0.041 (3)	0.041 (3)	0.055 (3)	0.000 (2)	0.005 (2)	−0.002 (2)
C62	0.068 (4)	0.038 (3)	0.065 (3)	0.000 (2)	0.001 (3)	0.005 (3)
C63	0.072 (4)	0.060 (3)	0.048 (3)	0.002 (3)	−0.003 (3)	0.003 (3)
C64	0.077 (4)	0.042 (3)	0.056 (3)	0.007 (3)	0.004 (3)	−0.001 (3)
C65	0.091 (5)	0.040 (3)	0.057 (3)	0.006 (3)	0.000 (3)	−0.003 (3)
C66	0.068 (4)	0.047 (3)	0.051 (3)	0.003 (3)	0.000 (3)	0.007 (2)
C67	0.041 (3)	0.041 (3)	0.067 (4)	0.001 (2)	0.003 (2)	0.004 (3)
O73	0.125 (6)	0.246 (9)	0.106 (5)	0.083 (6)	0.012 (4)	0.027 (5)
O71	0.131 (6)	0.113 (5)	0.160 (6)	−0.001 (4)	−0.008 (4)	0.019 (4)
O72	0.098 (5)	0.127 (5)	0.148 (6)	−0.019 (4)	−0.044 (4)	−0.001 (4)
C71	0.056 (5)	0.095 (6)	0.160 (9)	−0.008 (4)	−0.017 (5)	0.029 (6)
C72	0.087 (6)	0.109 (7)	0.135 (8)	−0.004 (5)	−0.017 (5)	−0.013 (6)
C73	0.102 (7)	0.127 (7)	0.132 (8)	0.027 (6)	−0.026 (6)	−0.043 (7)
C74	0.078 (6)	0.115 (7)	0.137 (8)	0.009 (5)	0.006 (5)	−0.001 (7)
C75	0.075 (6)	0.131 (8)	0.147 (9)	−0.004 (5)	0.019 (5)	−0.037 (7)
C76	0.088 (6)	0.154 (9)	0.103 (7)	−0.018 (6)	0.027 (5)	−0.010 (7)
C77	0.066 (5)	0.133 (8)	0.128 (8)	−0.020 (5)	−0.019 (5)	0.015 (7)
O1W	0.055 (2)	0.078 (3)	0.058 (2)	−0.0063 (19)	0.0042 (18)	−0.0151 (19)
O2W	0.090 (3)	0.066 (2)	0.066 (2)	−0.008 (2)	0.017 (2)	0.001 (2)
O3W	0.089 (3)	0.055 (2)	0.089 (3)	0.007 (2)	0.001 (2)	−0.011 (2)
O4W	0.112 (4)	0.069 (3)	0.075 (3)	0.035 (3)	0.009 (3)	0.002 (2)
O5W	0.079 (3)	0.077 (3)	0.120 (4)	−0.003 (2)	0.010 (3)	−0.004 (3)
O6W	0.082 (4)	0.095 (3)	0.134 (4)	0.008 (3)	−0.004 (3)	−0.020 (3)
O7W	0.136 (6)	0.208 (7)	0.115 (5)	−0.034 (5)	−0.030 (4)	−0.005 (5)

### Geometric parameters (Å, º)

**Table:**

Cu1—O53	1.941 (3)	C25—C26	1.406 (8)
Cu1—N1	2.002 (3)	C25—H25	0.9300
Cu1—N2	2.051 (4)	C26—C27	1.349 (8)
Cu1—N3	2.027 (3)	C26—H26	0.9300
Cu1—N4	2.158 (4)	C27—C28	1.394 (8)
Cu2—O51	1.979 (3)	C27—H27	0.9300
Cu2—N5	2.010 (4)	C28—C36	1.399 (7)
Cu2—N6	2.197 (4)	C28—C29	1.435 (8)
Cu2—N7	2.013 (4)	C29—C30	1.349 (9)
Cu2—N8	2.079 (4)	C29—H29	0.9300
O51—C57	1.259 (6)	C30—C31	1.422 (9)
O52—C57	1.230 (6)	C30—H30	0.9300
O53—C54	1.320 (5)	C31—C35	1.397 (7)
C51—C52	1.392 (7)	C31—C32	1.399 (9)
C51—C56	1.392 (7)	C32—C33	1.361 (10)
C51—C57	1.514 (7)	C32—H32	0.9300
C52—C53	1.375 (7)	C33—C34	1.384 (8)
C52—H52	0.9300	C33—H33	0.9300
C53—C54	1.402 (7)	C34—H34	0.9300
C53—H53	0.9300	C35—C36	1.439 (7)
C54—C55	1.427 (7)	C37—C38	1.384 (8)
C55—C56	1.393 (7)	C37—H37	0.9300
C55—H55	0.9300	C38—C39	1.359 (8)
C56—H56	0.9300	C38—H38	0.9300
N1—C1	1.327 (6)	C39—C40	1.406 (8)
N1—C12	1.356 (6)	C39—H39	0.9300
N2—C10	1.323 (6)	C40—C48	1.405 (7)
N2—C11	1.363 (5)	C40—C41	1.423 (8)
N3—C13	1.324 (6)	C41—C42	1.336 (9)
N3—C24	1.354 (6)	C41—H41	0.9300
N4—C22	1.324 (6)	C42—C43	1.450 (8)
N4—C23	1.361 (5)	C42—H42	0.9300
N5—C25	1.320 (6)	C43—C44	1.384 (8)
N5—C36	1.369 (6)	C43—C47	1.403 (7)
N6—C34	1.313 (7)	C44—C45	1.359 (9)
N6—C35	1.362 (7)	C44—H44	0.9300
N7—C37	1.323 (6)	C45—C46	1.395 (8)
N7—C48	1.367 (6)	C45—H45	0.9300
N8—C46	1.316 (6)	C46—H46	0.9300
N8—C47	1.360 (6)	C47—C48	1.434 (7)
C1—C2	1.393 (6)	O61—C67	1.246 (6)
C1—H1	0.9300	O62—C67	1.263 (6)
C2—C3	1.354 (7)	O63—C64	1.357 (6)
C2—H2	0.9300	O63—H63	0.8200
C3—C4	1.406 (7)	C61—C66	1.379 (7)
C3—H3	0.9300	C61—C62	1.399 (7)
C4—C12	1.396 (6)	C61—C67	1.500 (7)
C4—C5	1.430 (7)	C62—C63	1.365 (7)
C5—C6	1.345 (8)	C62—H62	0.9300
C5—H5	0.9300	C63—C64	1.392 (7)
C6—C7	1.436 (7)	C63—H63A	0.9300
C6—H6	0.9300	C64—C65	1.372 (7)
C7—C11	1.396 (6)	C65—C66	1.375 (7)
C7—C8	1.402 (7)	C65—H65	0.9300
C8—C9	1.359 (8)	C66—H66	0.9300
C8—H8	0.9300	O73—C74	1.363 (11)
C9—C10	1.397 (8)	O73—H73	0.8200
C9—H9	0.9300	O71—C77	1.294 (12)
C10—H10	0.9300	O72—C77	1.321 (12)
C11—C12	1.439 (6)	C71—C72	1.339 (11)
C13—C14	1.396 (7)	C71—C76	1.410 (12)
C13—H13	0.9300	C71—C77	1.487 (13)
C14—C15	1.352 (8)	C72—C73	1.412 (13)
C14—H14	0.9300	C72—H72	0.9300
C15—C16	1.382 (8)	C73—C74	1.397 (12)
C15—H15	0.9300	C73—H71	0.9300
C16—C24	1.417 (6)	C74—C75	1.403 (13)
C16—C17	1.445 (8)	C75—C76	1.454 (13)
C17—C18	1.341 (9)	C75—H75	0.9300
C17—H17	0.9300	C76—H76	0.9300
C18—C19	1.432 (8)	O1W—H1A	0.97
C18—H18	0.9300	O1W—H1B	1.00
C19—C23	1.404 (7)	O2W—H2A	0.97
C19—C20	1.410 (8)	O2W—H2B	0.83
C20—C21	1.342 (8)	O3W—H3A	1.00
C20—H20	0.9300	O3W—H3B	1.00
C21—C22	1.388 (7)	O4W—H4A	1.01
C21—H21	0.9300	O4W—H4B	0.86
C22—H22	0.9300	O5W—H5A	0.96
C23—C24	1.437 (7)	O5W—H5B	0.97
			
N1—Cu1—N3	176.67 (15)	N4—C22—C21	123.3 (5)
O53—Cu1—N2	155.19 (14)	N4—C22—H22	118.3
O53—Cu1—N4	101.39 (14)	C21—C22—H22	118.3
N2—Cu1—N4	103.05 (15)	N4—C23—C19	123.0 (4)
O53—Cu1—N1	89.92 (14)	N4—C23—C24	116.7 (4)
O53—Cu1—N3	93.37 (14)	C19—C23—C24	120.3 (4)
N1—Cu1—N2	81.74 (14)	N3—C24—C16	122.0 (4)
N3—Cu1—N2	95.05 (15)	N3—C24—C23	118.1 (4)
N1—Cu1—N4	100.08 (14)	C16—C24—C23	119.9 (4)
N3—Cu1—N4	79.74 (15)	N5—C25—C26	122.8 (5)
N5—Cu2—N7	173.07 (16)	N5—C25—H25	118.6
O51—Cu2—N5	96.10 (15)	C26—C25—H25	118.6
O51—Cu2—N7	89.77 (15)	C27—C26—C25	119.5 (6)
O51—Cu2—N8	152.80 (15)	C27—C26—H26	120.3
N5—Cu2—N8	94.96 (16)	C25—C26—H26	120.3
N7—Cu2—N8	81.32 (17)	C26—C27—C28	119.7 (5)
O51—Cu2—N6	101.29 (16)	C26—C27—H27	120.1
N5—Cu2—N6	79.62 (16)	C28—C27—H27	120.1
N7—Cu2—N6	95.64 (16)	C27—C28—C36	117.9 (5)
N8—Cu2—N6	105.12 (16)	C27—C28—C29	123.7 (5)
C57—O51—Cu2	112.6 (3)	C36—C28—C29	118.5 (6)
C54—O53—Cu1	122.0 (3)	C30—C29—C28	121.1 (6)
C52—C51—C56	117.5 (4)	C30—C29—H29	119.5
C52—C51—C57	120.7 (4)	C28—C29—H29	119.5
C56—C51—C57	121.7 (4)	C29—C30—C31	121.6 (5)
C53—C52—C51	122.2 (4)	C29—C30—H30	119.2
C53—C52—H52	118.9	C31—C30—H30	119.2
C51—C52—H52	118.9	C35—C31—C32	117.0 (6)
C52—C53—C54	121.0 (4)	C35—C31—C30	118.9 (6)
C52—C53—H53	119.5	C32—C31—C30	124.1 (6)
C54—C53—H53	119.5	C33—C32—C31	120.0 (6)
O53—C54—C53	124.2 (4)	C33—C32—H32	120.0
O53—C54—C55	118.6 (4)	C31—C32—H32	120.0
C53—C54—C55	117.2 (4)	C32—C33—C34	118.6 (6)
C56—C55—C54	120.2 (4)	C32—C33—H33	120.7
C56—C55—H55	119.9	C34—C33—H33	120.7
C54—C55—H55	119.9	N6—C34—C33	124.1 (6)
C51—C56—C55	121.7 (5)	N6—C34—H34	117.9
C51—C56—H56	119.1	C33—C34—H34	117.9
C55—C56—H56	119.1	N6—C35—C31	122.8 (5)
O52—C57—O51	124.3 (5)	N6—C35—C36	117.3 (4)
O52—C57—C51	119.7 (5)	C31—C35—C36	119.8 (5)
O51—C57—C51	115.9 (4)	N5—C36—C28	122.4 (5)
C1—N1—C12	118.2 (4)	N5—C36—C35	117.5 (4)
C1—N1—Cu1	128.2 (3)	C28—C36—C35	120.1 (5)
C12—N1—Cu1	113.5 (3)	N7—C37—C38	122.2 (5)
C10—N2—C11	117.5 (4)	N7—C37—H37	118.9
C10—N2—Cu1	130.8 (3)	C38—C37—H37	118.9
C11—N2—Cu1	111.7 (3)	C39—C38—C37	120.5 (6)
C13—N3—C24	118.5 (4)	C39—C38—H38	119.8
C13—N3—Cu1	127.0 (3)	C37—C38—H38	119.8
C24—N3—Cu1	114.4 (3)	C38—C39—C40	119.6 (5)
C22—N4—C23	117.1 (4)	C38—C39—H39	120.2
C22—N4—Cu1	132.1 (3)	C40—C39—H39	120.2
C23—N4—Cu1	110.6 (3)	C48—C40—C39	116.6 (5)
C25—N5—C36	117.7 (4)	C48—C40—C41	118.5 (5)
C25—N5—Cu2	126.7 (3)	C39—C40—C41	124.9 (5)
C36—N5—Cu2	115.6 (3)	C42—C41—C40	121.2 (5)
C34—N6—C35	117.4 (5)	C42—C41—H41	119.4
C34—N6—Cu2	132.7 (4)	C40—C41—H41	119.4
C35—N6—Cu2	109.8 (3)	C41—C42—C43	122.4 (5)
C37—N7—C48	118.3 (4)	C41—C42—H42	118.8
C37—N7—Cu2	128.3 (4)	C43—C42—H42	118.8
C48—N7—Cu2	113.4 (3)	C44—C43—C47	117.3 (5)
C46—N8—C47	117.5 (4)	C44—C43—C42	125.5 (5)
C46—N8—Cu2	131.0 (4)	C47—C43—C42	117.2 (5)
C47—N8—Cu2	111.5 (3)	C45—C44—C43	120.2 (5)
N1—C1—C2	122.1 (4)	C45—C44—H44	119.9
N1—C1—H1	119.0	C43—C44—H44	119.9
C2—C1—H1	119.0	C44—C45—C46	118.8 (6)
C3—C2—C1	119.8 (5)	C44—C45—H45	120.6
C3—C2—H2	120.1	C46—C45—H45	120.6
C1—C2—H2	120.1	N8—C46—C45	123.4 (6)
C2—C3—C4	120.1 (4)	N8—C46—H46	118.3
C2—C3—H3	119.9	C45—C46—H46	118.3
C4—C3—H3	119.9	N8—C47—C43	122.8 (5)
C12—C4—C3	116.5 (4)	N8—C47—C48	116.9 (4)
C12—C4—C5	118.3 (5)	C43—C47—C48	120.3 (5)
C3—C4—C5	125.2 (4)	N7—C48—C40	122.8 (5)
C6—C5—C4	121.7 (5)	N7—C48—C47	116.8 (4)
C6—C5—H5	119.1	C40—C48—C47	120.3 (5)
C4—C5—H5	119.1	C64—O63—H63	109.5
C5—C6—C7	121.2 (5)	C66—C61—C62	117.5 (4)
C5—C6—H6	119.4	C66—C61—C67	120.5 (5)
C7—C6—H6	119.4	C62—C61—C67	122.0 (4)
C11—C7—C8	117.5 (5)	C63—C62—C61	122.0 (5)
C11—C7—C6	118.3 (5)	C63—C62—H62	119.0
C8—C7—C6	124.2 (5)	C61—C62—H62	119.0
C9—C8—C7	118.8 (5)	C62—C63—C64	119.5 (5)
C9—C8—H8	120.6	C62—C63—H63A	120.3
C7—C8—H8	120.6	C64—C63—H63A	120.3
C8—C9—C10	120.4 (5)	O63—C64—C65	118.8 (5)
C8—C9—H9	119.8	O63—C64—C63	122.0 (5)
C10—C9—H9	119.8	C65—C64—C63	119.1 (5)
N2—C10—C9	122.4 (5)	C64—C65—C66	121.0 (5)
N2—C10—H10	118.8	C64—C65—H65	119.5
C9—C10—H10	118.8	C66—C65—H65	119.5
N2—C11—C7	123.4 (4)	C65—C66—C61	120.9 (5)
N2—C11—C12	116.4 (4)	C65—C66—H66	119.6
C7—C11—C12	120.2 (4)	C61—C66—H66	119.6
N1—C12—C4	123.3 (4)	O61—C67—O62	124.3 (5)
N1—C12—C11	116.6 (4)	O61—C67—C61	119.5 (4)
C4—C12—C11	120.1 (4)	O62—C67—C61	116.2 (5)
N3—C13—C14	122.5 (5)	C74—O73—H73	109.5
N3—C13—H13	118.8	C72—C71—C76	120.4 (10)
C14—C13—H13	118.8	C72—C71—C77	120.5 (11)
C15—C14—C13	119.2 (5)	C76—C71—C77	119.0 (9)
C15—C14—H14	120.4	C71—C72—C73	121.9 (10)
C13—C14—H14	120.4	C71—C72—H72	119.0
C14—C15—C16	120.7 (5)	C73—C72—H72	119.0
C14—C15—H15	119.7	C74—C73—C72	120.2 (9)
C16—C15—H15	119.7	C74—C73—H71	119.9
C15—C16—C24	117.1 (5)	C72—C73—H71	119.9
C15—C16—C17	125.4 (5)	O73—C74—C73	124.4 (10)
C24—C16—C17	117.5 (5)	O73—C74—C75	116.3 (10)
C18—C17—C16	122.4 (5)	C73—C74—C75	119.1 (10)
C18—C17—H17	118.8	C74—C75—C76	119.5 (9)
C16—C17—H17	118.8	C74—C75—H75	120.2
C17—C18—C19	121.0 (5)	C76—C75—H75	120.2
C17—C18—H18	119.5	C71—C76—C75	118.7 (10)
C19—C18—H18	119.5	C71—C76—H76	120.7
C23—C19—C20	117.0 (5)	C75—C76—H76	120.7
C23—C19—C18	118.8 (5)	O71—C77—O72	125.2 (9)
C20—C19—C18	124.1 (5)	O71—C77—C71	115.3 (10)
C21—C20—C19	119.3 (5)	O72—C77—C71	119.4 (10)
C21—C20—H20	120.3	H1A—O1W—H1B	103
C19—C20—H20	120.3	H2A—O2W—H2B	121
C20—C21—C22	120.1 (5)	H3A—O3W—H3B	102
C20—C21—H21	119.9	H4A—O4W—H4B	107
C22—C21—H21	119.9	H5A—O5W—H5B	114
			
C56—C51—C52—C53	0.5 (7)	C26—C27—C28—C29	178.1 (5)
C57—C51—C52—C53	−174.7 (4)	C27—C28—C29—C30	−177.7 (5)
C51—C52—C53—C54	3.8 (7)	C36—C28—C29—C30	1.2 (8)
Cu1—O53—C54—C53	12.0 (5)	C28—C29—C30—C31	−2.5 (9)
Cu1—O53—C54—C55	−170.4 (3)	C29—C30—C31—C35	1.9 (8)
C52—C53—C54—O53	172.1 (4)	C29—C30—C31—C32	−179.4 (6)
C52—C53—C54—C55	−5.6 (6)	C35—C31—C32—C33	−0.9 (9)
O53—C54—C55—C56	−174.6 (4)	C30—C31—C32—C33	−179.7 (6)
C53—C54—C55—C56	3.2 (6)	C31—C32—C33—C34	−0.2 (10)
C52—C51—C56—C55	−2.9 (7)	C35—N6—C34—C33	−0.3 (9)
C57—C51—C56—C55	172.2 (4)	Cu2—N6—C34—C33	174.4 (5)
C54—C55—C56—C51	1.0 (7)	C32—C33—C34—N6	0.9 (10)
Cu2—O51—C57—O52	6.4 (6)	C34—N6—C35—C31	−0.9 (7)
Cu2—O51—C57—C51	−171.7 (3)	Cu2—N6—C35—C31	−176.8 (4)
C52—C51—C57—O52	22.8 (6)	C34—N6—C35—C36	179.5 (5)
C56—C51—C57—O52	−152.2 (5)	Cu2—N6—C35—C36	3.6 (5)
C52—C51—C57—O51	−158.9 (4)	C32—C31—C35—N6	1.6 (7)
C56—C51—C57—O51	26.1 (6)	C30—C31—C35—N6	−179.6 (5)
C12—N1—C1—C2	0.8 (7)	C32—C31—C35—C36	−178.8 (5)
Cu1—N1—C1—C2	179.3 (3)	C30—C31—C35—C36	0.0 (7)
N1—C1—C2—C3	−0.2 (7)	C25—N5—C36—C28	−0.4 (7)
C1—C2—C3—C4	−0.1 (8)	Cu2—N5—C36—C28	179.0 (4)
C2—C3—C4—C12	−0.1 (7)	C25—N5—C36—C35	−179.3 (4)
C2—C3—C4—C5	177.8 (5)	Cu2—N5—C36—C35	0.2 (5)
C12—C4—C5—C6	0.3 (8)	C27—C28—C36—N5	0.8 (7)
C3—C4—C5—C6	−177.6 (5)	C29—C28—C36—N5	−178.2 (4)
C4—C5—C6—C7	−2.9 (9)	C27—C28—C36—C35	179.7 (4)
C5—C6—C7—C11	3.4 (8)	C29—C28—C36—C35	0.7 (7)
C5—C6—C7—C8	−175.8 (5)	N6—C35—C36—N5	−2.7 (6)
C11—C7—C8—C9	0.1 (8)	C31—C35—C36—N5	177.7 (4)
C6—C7—C8—C9	179.3 (5)	N6—C35—C36—C28	178.4 (4)
C7—C8—C9—C10	0.7 (9)	C31—C35—C36—C28	−1.2 (7)
C11—N2—C10—C9	−0.6 (8)	C48—N7—C37—C38	0.0 (8)
Cu1—N2—C10—C9	178.5 (4)	Cu2—N7—C37—C38	−179.3 (4)
C8—C9—C10—N2	−0.5 (9)	N7—C37—C38—C39	0.7 (9)
C10—N2—C11—C7	1.5 (7)	C37—C38—C39—C40	0.1 (9)
Cu1—N2—C11—C7	−177.8 (4)	C38—C39—C40—C48	−1.5 (8)
C10—N2—C11—C12	−177.7 (4)	C38—C39—C40—C41	177.8 (5)
Cu1—N2—C11—C12	3.0 (5)	C48—C40—C41—C42	−3.6 (8)
C8—C7—C11—N2	−1.2 (7)	C39—C40—C41—C42	177.2 (6)
C6—C7—C11—N2	179.5 (4)	C40—C41—C42—C43	2.6 (9)
C8—C7—C11—C12	177.9 (4)	C41—C42—C43—C44	178.2 (6)
C6—C7—C11—C12	−1.3 (7)	C41—C42—C43—C47	−0.1 (8)
C1—N1—C12—C4	−1.0 (7)	C47—C43—C44—C45	1.4 (9)
Cu1—N1—C12—C4	−179.8 (3)	C42—C43—C44—C45	−176.9 (6)
C1—N1—C12—C11	179.8 (4)	C43—C44—C45—C46	0.0 (9)
Cu1—N1—C12—C11	1.1 (5)	C47—N8—C46—C45	−0.1 (8)
C3—C4—C12—N1	0.7 (7)	Cu2—N8—C46—C45	−179.2 (4)
C5—C4—C12—N1	−177.4 (4)	C44—C45—C46—N8	−0.7 (9)
C3—C4—C12—C11	179.9 (4)	C46—N8—C47—C43	1.6 (7)
C5—C4—C12—C11	1.8 (7)	Cu2—N8—C47—C43	−179.1 (4)
N2—C11—C12—N1	−2.8 (6)	C46—N8—C47—C48	179.2 (4)
C7—C11—C12—N1	178.0 (4)	Cu2—N8—C47—C48	−1.5 (5)
N2—C11—C12—C4	178.0 (4)	C44—C43—C47—N8	−2.3 (7)
C7—C11—C12—C4	−1.2 (7)	C42—C43—C47—N8	176.1 (5)
C24—N3—C13—C14	0.2 (8)	C44—C43—C47—C48	−179.7 (5)
Cu1—N3—C13—C14	−175.7 (4)	C42—C43—C47—C48	−1.4 (7)
N3—C13—C14—C15	0.2 (8)	C37—N7—C48—C40	−1.5 (7)
C13—C14—C15—C16	0.9 (9)	Cu2—N7—C48—C40	177.9 (4)
C14—C15—C16—C24	−2.4 (8)	C37—N7—C48—C47	179.3 (4)
C14—C15—C16—C17	178.1 (5)	Cu2—N7—C48—C47	−1.3 (5)
C15—C16—C17—C18	178.6 (6)	C39—C40—C48—N7	2.2 (7)
C24—C16—C17—C18	−0.9 (8)	C41—C40—C48—N7	−177.1 (4)
C16—C17—C18—C19	−1.9 (9)	C39—C40—C48—C47	−178.6 (4)
C17—C18—C19—C23	3.8 (8)	C41—C40—C48—C47	2.1 (7)
C17—C18—C19—C20	−176.9 (6)	N8—C47—C48—N7	1.9 (6)
C23—C19—C20—C21	0.7 (8)	C43—C47—C48—N7	179.6 (4)
C18—C19—C20—C21	−178.5 (5)	N8—C47—C48—C40	−177.3 (4)
C19—C20—C21—C22	0.3 (9)	C43—C47—C48—C40	0.3 (7)
C23—N4—C22—C21	0.1 (7)	C66—C61—C62—C63	−1.7 (8)
Cu1—N4—C22—C21	174.9 (4)	C67—C61—C62—C63	178.0 (5)
C20—C21—C22—N4	−0.7 (9)	C61—C62—C63—C64	1.0 (9)
C22—N4—C23—C19	1.0 (7)	C62—C63—C64—O63	−179.5 (6)
Cu1—N4—C23—C19	−174.9 (4)	C62—C63—C64—C65	0.3 (9)
C22—N4—C23—C24	−178.1 (4)	O63—C64—C65—C66	178.9 (6)
Cu1—N4—C23—C24	6.0 (5)	C63—C64—C65—C66	−0.9 (9)
C20—C19—C23—N4	−1.4 (7)	C64—C65—C66—C61	0.2 (9)
C18—C19—C23—N4	177.9 (5)	C62—C61—C66—C65	1.0 (8)
C20—C19—C23—C24	177.7 (5)	C67—C61—C66—C65	−178.6 (5)
C18—C19—C23—C24	−3.0 (7)	C66—C61—C67—O61	9.9 (8)
C13—N3—C24—C16	−1.8 (7)	C62—C61—C67—O61	−169.7 (5)
Cu1—N3—C24—C16	174.6 (3)	C66—C61—C67—O62	−171.1 (5)
C13—N3—C24—C23	179.0 (4)	C62—C61—C67—O62	9.2 (8)
Cu1—N3—C24—C23	−4.6 (5)	C76—C71—C72—C73	2.7 (13)
C15—C16—C24—N3	2.9 (7)	C77—C71—C72—C73	178.8 (8)
C17—C16—C24—N3	−177.6 (4)	C71—C72—C73—C74	−1.9 (14)
C15—C16—C24—C23	−177.9 (5)	C72—C73—C74—O73	−176.2 (8)
C17—C16—C24—C23	1.6 (7)	C72—C73—C74—C75	−1.7 (14)
N4—C23—C24—N3	−1.3 (6)	O73—C74—C75—C76	179.2 (8)
C19—C23—C24—N3	179.6 (4)	C73—C74—C75—C76	4.3 (13)
N4—C23—C24—C16	179.5 (4)	C72—C71—C76—C75	0.0 (12)
C19—C23—C24—C16	0.3 (7)	C77—C71—C76—C75	−176.2 (7)
C36—N5—C25—C26	0.0 (7)	C74—C75—C76—C71	−3.5 (12)
Cu2—N5—C25—C26	−179.4 (4)	C72—C71—C77—O71	174.9 (8)
N5—C25—C26—C27	−0.1 (9)	C76—C71—C77—O71	−8.9 (11)
C25—C26—C27—C28	0.5 (8)	C72—C71—C77—O72	−7.1 (12)
C26—C27—C28—C36	−0.9 (8)	C76—C71—C77—O72	169.1 (8)

### Hydrogen-bond geometry (Å, º)

**Table:**

*D*—H···*A*	*D*—H	H···*A*	*D*···*A*	*D*—H···*A*
O1*W*—H1*A*···O53	0.97	1.90	2.819 (5)	157
O1*W*—H1*B*···O61^i^	1.00	1.76	2.758 (5)	169
O2*W*—H2*A*···O61^i^	0.97	1.75	2.708 (5)	169
O2*W*—H2*B*···O4*W*^ii^	0.83	2.04	2.843 (6)	160
O3*W*—H3*A*···O62^iii^	1.00	1.69	2.685 (5)	172
O3*W*—H3*B*···O62	1.00	1.84	2.731 (7)	147
O4*W*—H4*A*···O1*W*	1.01	1.79	2.756 (6)	160
O4*W*—H4*B*···O3*W*	0.86	1.94	2.750 (7)	157
O5*W*—H5*A*···O72	0.96	1.87	2.818 (8)	169
O5*W*—H5*B*···O6*W*^iv^	0.97	1.84	2.779 (7)	164
O73—H73···O8*W*	0.82	2.15	2.88 (2)	148
O63—H63···O2*W*	0.82	1.85	2.638 (5)	161
C3—H3···O1*W*^i^	0.93	2.59	3.240 (6)	127
C8—H8···O6*W*	0.93	2.48	3.371 (8)	162
C14—H14···O72	0.93	2.50	3.388 (10)	159
C21—H21···O4*W*^ii^	0.93	2.58	3.230 (8)	128
C25—H25···O52	0.93	2.47	3.033 (6)	119
C33—H33···O62^v^	0.93	2.55	3.247 (8)	132
C38—H38···O61^iii^	0.93	2.37	3.298 (8)	172
C65—H65···O63^vi^	0.93	2.49	3.410 (6)	172
C73—H71···O7*W*^iv^	0.93	2.58	3.413 (13)	149

Symmetry codes: (i) *x*, −*y*+1/2, *z*+1/2; (ii) −*x*+1, *y*+1/2, −*z*+1/2; (iii) −*x*+1, −*y*, −*z*; (iv) *x*, −*y*+1/2, *z*−1/2; (v) −*x*+1, *y*−1/2, −*z*+1/2; (vi) −*x*+1, −*y*+1, −*z*.
